# Seeking care from a traditional healer after injury in Sudan: an exploratory cross-sectional analysis

**DOI:** 10.1093/inthealth/ihz063

**Published:** 2019-07-23

**Authors:** Safa Abdalla, Muna Abdel Aziz, Igbal Basheir, Sudan Household Health Survey 2010 national management team

**Affiliations:** Sudan Health Consultancy, Solihull, UK; Sudan Health Consultancy, Solihull, UK; Federal Ministry of Health, P.O.Box 303, Khartoum, Sudan

**Keywords:** injury care, Sudan, traditional healers

## Abstract

**Background:**

Seeking care from traditional healers for injury is a common practice in low- and middle-income countries, including Sudan. As little is known about specific patterns of the practice in the country, we aimed to investigate associated factors and the role of professional injury care availability.

**Methods:**

We used Sudan Household Health Survey 2010 data from a national stratified multistage cluster sample of 15 000 households. A multivariable Poisson regression (PR) model with robust variance was used to test potential associations of receiving care from a traditional healer in the first week after injury with age, gender, urban/rural residence, wealth index, educational attainment, cause of injury, time of injury occurrence and state-level injury-care bed density.

**Results:**

Of 1432 injured participants who sought some form of healthcare, 38% received care from a traditional healer. A significant negative association was found with educational attainment, age and wealth. The association between injury-care bed density and receiving care from a traditional healer was consistently evident only when the injury was caused by a road traffic accident (PR = 0.90, 95% CI 0.85 to 0.96).

**Conclusions:**

Merely increasing the affordability or availability of injury care facilities may not impact reliance on traditional healers for all causes of injury. Therefore, injury care policies need to consider the role of traditional healers as part of the healthcare system.

## Introduction

Seeking care from a traditional healer is a common practice in many low- and middle-income countries (LMICs).^1,2^ Studies from Kenya, Nigeria and Ghana showed that in those communities, poor people are more likely to go to traditional healers for the treatment of sickness or injury rather than people who are more affluent.^3,4^ Unlike in developed countries, traditional/complementary medicine in developing countries is not well regulated, and the practice was documented to have undesirable consequences, directly due to inappropriate treatments or indirectly due to a delay in seeking appropriate healthcare from formal sources.^5–7^ Nevertheless, it remains deeply grounded in many cultures due to the trust and high regard traditionally afforded to village or tribe healers, in addition to them being viewed as more affordable, accessible, empathetic and patient-centred than formal healthcare-givers.^8–10^ For those reasons there has been widespread acknowledgement of the potential role of traditional healers in supporting healthcare delivery and the need for promoting the safe and effective use of traditional medicine.^1,11,12^

The sparse literature on attending traditional healers for injury care shows that seeking care from a traditional healer is significant, consisting of up to 50% of those injured by snakebite in a subdistrict of South Africa, 20% of those who sustained an eye injury in a rural southern India population and 21% of those with a burn injury in Sierra Leone.^6,13,14^ Yet traditional healers and their role in injury care do not usually feature in conversations about injury care in LMICs. Understandably, the burden of life-threatening serious injuries demands a focus on the development of formal professional emergency care. However, inappropriate management or a delay in appropriate management of non-life threatening injuries can result in significant disability.^5,7^ Given documented socioeconomic differentials, this could potentially result in wide socioeconomic differentials in disability after injury.^15^

Sudan is an LMIC where reliance on traditional healers for injury care has been documented. Data from the 2010 Sudan Household Health Survey (SHHS) showed that about one third of injured people had received care from a traditional healer in the first week after injury.^16^ In Khartoum, a predominantly urban state with a high concentration of health services, people of a lower socioeconomic status were less likely to use formal health services after injury than those with a higher socioeconomic status.^17^ Patterns could be different in other parts of the country that have different health service availability or which are predominantly rural.

Uncovering national trends with a focus on traditional healer use is needed to inform the discourse on the role of traditional healers in injury care, and the possible directions for and implications of injury care-related policies in Sudan, such as integration of traditional healers and equity in healthcare distribution. This is highly relevant in a country where a shortage in healthcare workers and inequity in their distribution prevails.^18^ Therefore, in this paper we sought to investigate the factors associated with seeking care from a traditional healer after injury in Sudan and the role of the availability of professional injury care.

## Methods

### Setting

Sudan has a population of 30 million, 70% of which lives in rural areas. Slightly less than half the population is aged <15 y. Under-five mortality has been declining, amounting to 78 per 1000 live births in 2010.^16^ The country is now divided into 18 states (previously 15), of which only one—Khartoum—is mostly urban (80%). Professional, managerial and elementary occupations are common in urban Sudan while agriculture predominates in rural areas.^19^

### Data

For data on sociodemographic and injury variables, we used the SHHS 2010. Details of the survey methodology have been published elsewhere.^16^ The survey used a multistage cluster sample of 15 000 households stratified by state and by urban-rural distribution of the population in each state. The sample size was based on an estimation of key women and child health-related indicators (such as infant mortality and prevalence of malnutrition) at state level, balanced by costs and logistics. The final sample size was 83 510, representing an individual response rate of 90%. The survey was approved by the Federal Ministry of Health Research Ethics Committee and respondents provided informed consent to participate. A questionnaire administered by trained data collectors in face-to-face interviews was used to collect data on demographic, social and health variables, including an injury module. The module asked each member of the household whether they had sustained an injury in the 12-mo period preceding the survey and, if so, the time, cause, type of healthcare received and disability related to the most recent injury. We included participants who were reported to have suffered an injury in the 12 mo preceding the survey and sought some form of healthcare.

For data on professional injury care availability, we used the total number of beds allocated to emergency, orthopaedic and general surgery departments (the departments concerned with injury care),^20^ and calculated injury-care bed density as beds to 100 000 population ratio for each state using 2008 census state population estimates.^21^ The variable can capture the number as well as the size of health facilities with professional injury care services. We used a state-level variable because it is an important point of intervention and indicator in health workforce policies which aim to achieve equitable distribution, particularly in the context of injury care.

### Statistical analysis

We carried out complete case analysis, first with descriptive statistics and cross-tabulations for general univariable patterns of use of traditional healers by age, gender, area of residence (urban/rural), wealth index, educational level, cause of injury and state-level injury-care bed to population ratio, accounting for sampling weights and complex survey design. For illustrative purposes and only in the descriptive statistics, age was classified into five groups and wealth index score into quintiles. They were used in their continuous forms in all other analyses to avoid the loss of information that results from using the aggregated forms.

The decision to seek formal healthcare instead of a traditional healer could be influenced by the perception of the severity of injury.^17^ Since we did not have a measure of injury severity in the SHHS we accounted for this in a different way. Memory decay is known to affect the recall of injuries and differentially filters out minor injuries that did not occur closer to the time of the interview.^22^ Based on evidence of memory decay in our data,^23^ we included the time the injury was reported to have occurred (dichotomised) as a possible confounding or effect-modifying variable in the regression analysis. Table 1 provides detailed descriptions of all the variables included.

**Table 1.** Description of independent variables

Variable	Description
Age	Age in years
Gender	Male/female
Area of residence	Urban/rural
Wealth index	A continuous variable calculated with principal components analysis of a range of household assets and characteristics.^35^ The higher the score the wealthier the household is.
Educational level	None Primary Secondary or higher The construct of lower education would vary by age, as lower educational achievement is normal for children, whose healthcare decisions are determined by their immediate caregiver. To harmonise the construct across all ages, we used the mother’s education (or that of the head of the household when the mother was absent) for those aged <15 y.
Cause of injury	From the question: ‘What was the cause of the most recent injury?’ with the following response options: Road traffic accident Poisoning Falls Fire/hot substance (non-transport) Mechanical (non-transport) Animal bite or venom Assault Other
Injury-care bed density	Number of beds in each state allocated to emergency, orthopaedic and general surgery departments per 100 000 of the population.
Time of injury occurrence	From the question ‘When was the most recent injury?’ with four options: 4 wk ago, 5–12 wk ago, 13–24 wk ago and 25 wk to 12 mo ago. Dichotomised based on a large difference in average monthly percentage distribution of injuries reported over the recall period between the first time period and the others.^23^ 1 mo ago >1 mo ago
Seeking care from traditional healers	From the question ‘What type of healthcare did (name) receive during the first week of injury?’ with five responses: none, hospital inpatient, hospital outpatient, outpatient in health facility other than hospital or traditional healer. The variable was coded 1 if the traditional healer option was selected and 0 if any other option was selected.

We ran multivariable Poisson regression with robust variance estimation to model the effect of these variables on the probability of seeking care from a traditional healer in the first week and to estimate adjusted prevalence ratios. The robust variance accounts for the underdispersion that results from the application of Poisson regression to binomial data.^24,25^ This method has been recommended for the analysis of cross-sectional data to directly estimate prevalence ratios instead of ORs, which do not correctly represent the underlying prevalence ratio when the outcome is common,^25^ as is the case in our study. A prevalence ratio of >1 indicated that the variable was associated with a higher probability of use of traditional healers and a prevalence ratio of <1 indicated the opposite. All potential independent variables were initially included and then variables were removed sequentially, starting with the one with the highest p-value until only variables significant at the 0.05 level were retained (Supplementary Table 1a and 1b). We also ruled out a non-linear association with age by testing the statistical significance of quadratic and cubic terms for age.

It was also reasonable to suspect that there could be relevant interactions between those variables. We therefore proceeded by testing some interactions (further details and rationale are reported in Supplementary Table 2), keeping the statistically significant ones in the final model.

We also reran the analysis for the subset of those who were injured earlier than a month before the survey, where the proportion of minor injuries is expected to be much smaller. The analysis was carried out with R complex survey analysis package ‘survey’, R version 3.5.1, Vienna, Austria, accounting for the complex sampling design, sampling weights and the nesting of participants within the randomly selected households and clusters. States were not treated as an additional level because there was no sampling at that level.

## Results

**Figure 1:**
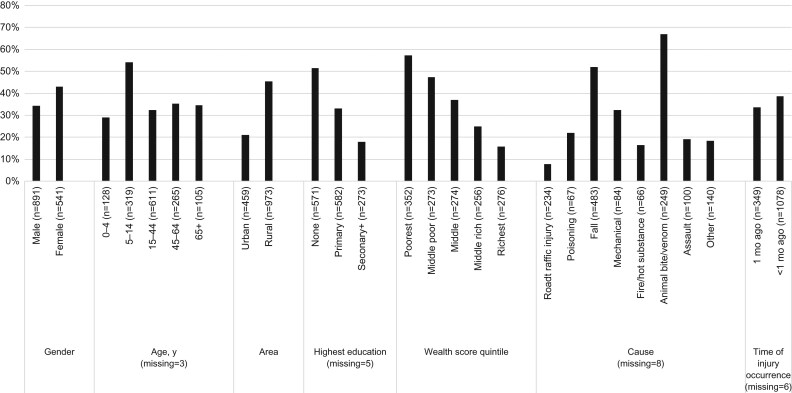
Weighted percentage of injured people who received care from a traditional healer in the first week by sociodemographic factors and injury-care factors.

**Figure 2:**
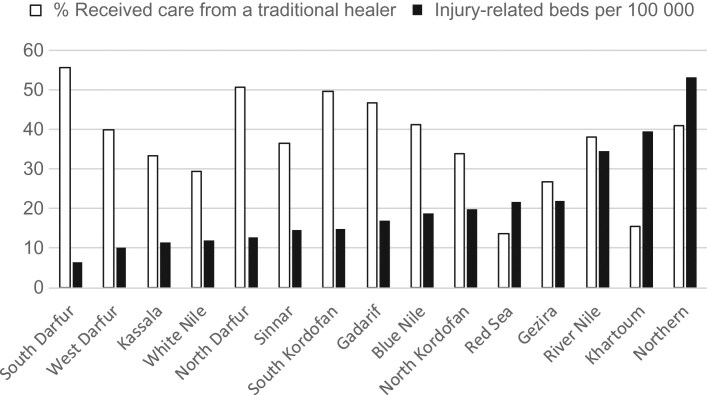
Weighted percentage of injured people in each state who received care from a traditional healer in the first week by state-level injury-care bed density.

Of 1432 participants who were injured and sought some form of healthcare outside the home, 38% received care from a traditional healer in the first week. Figure 1 shows the probability of receiving care from a traditional healer by key variables (unadjusted prevalence ratios are reported in Supplementary Table 3). Females, children aged 5–14 y, those living in rural areas, those with no education and lower wealth appeared to be more likely to receive care from a traditional healer, as were those injured by a fall, animal bite or venom or a mechanical cause. The highest percentage receiving care from a traditional healer was in South Darfur (56%), which also had the lowest injury bed-density rate (6 per 100 000) (Figure 2). Northern State had the highest injury bed-density rate (53 per 100 000) and also one of the highest proportions receiving care from a traditional healer after injury (41%).

In a multivariable model, higher educational level and increasing wealth were associated with a lower probability of receiving care from a traditional healer (Table 2). An increment in age of 5 y was associated with a 2% reduction in the probability of seeking care from a traditional healer. These results were stable when the analysis was limited to the subset of injuries that were reported to have occurred more than a month before the survey. Time of occurrence of injury and area of residence were not significant and there was no evidence that they influenced associations with other variables. Table 2 shows the final regression model with the only significant interaction found: between injury-care bed density in the state of residence of respondents and the cause of injury (p=0.03). At the lowest injury-care bed density, injury due to animal bite or venom was more than twice as likely to be seen by a traditional healer than a road traffic injury. With an increment of 1 injury-care bed per 100 000 people, the probability of receiving care from a traditional healer was 10% lower when the injury was caused by a road traffic accident and 5% lower when the injury was caused by fire or a hot substance (Table 3). However, only the results for road traffic injuries remained statistically significant after excluding injuries reported to have occurred in the first month.

**Table 2.** Adjusted prevalence ratio for care by traditional healer in the first week after injury, Sudan Household Health Survey 2010 (final model with significant variables)

	All time periods		Earlier than a month ago	
	Prevalence ratio (95% CI)	p-value	Prevalence ratio (95% CI)	p-value
Age (5-y increments)	0.98 (0.96 to 1.00)	0.047	0.97 (0.95 to 0.99)	0.003
Educational level (ref. = none)				
Primary	0.82 (0.70 to 0.97)	0.021	0.75 (0.61 to 0.93)	0.008
Secondary +	0.62 (0.45 to 0.86)	0.004	0.67 (0.46 to 0.98)	0.039
Wealth score	0.80 (0.69 to 0.93)	0.003	0.73 (0.62 to 0.87)	<0.001
Injury-care bed density^a^ centred on minimum^b^)	0.90 (0.85 to 0.96)	<0.001	0.91 (0.86 to 0.97)	0.004
Cause of injury^c^ (ref. = road traffic accident)				
Poisoning	1.06 (0.41 to 2.74)	0.904	0.97 (0.35 to 2.73)	0.955
Fall	1.83 (0.98 to 3.44)	0.060	1.54 (0.79 to 3.01)	0.210
Mechanical (non-transport)	1.07 (0.44 to 2.62)	0.876	0.99 (0.42 to 2.35)	0.983
Fire/hot substance (non-transport)	0.90 (0.38 to 2.14)	0.814	0.37 (0.12 to 1.19)	0.097
Animal bite/venom	2.15 (1.21 to 3.84)	0.010	1.86 (0.99 to 3.48)	0.055
Assault	0.66 (0.26 to 1.68)	0.386	0.46 (0.15 to 1.43)	0.179
Other	0.74 (0.27 to 2.04)	0.567	0.41 (0.14 to 1.22)	0.112
Interaction^d^ between injury-care bed density and:				
Poisoning	1.10 (1.02 to 1.19)	0.020	1.10 (1.02 to 1.19)	0.018
Fall	1.11 (1.04 to 1.18)	0.001	1.10 (1.03 to 1.17)	0.003
Mechanical (non-transport)	1.12 (1.02 to 1.23)	0.015	1.12 (1.03 to 1.21)	0.006
Fire/hot substance (non-transport)	1.06 (0.99 to 1.14)	0.106	1.09 (1.00 to 1.17)	0.042
Animal bite/venom	1.11 (1.05 to 1.18)	<0.001	1.10 (1.04 to 1.17)	0.002
Assault	1.12 (1.03 to 1.20)	0.005	1.11 (1.02 to 1.21)	0.013
Other	1.09 (0.98 to 1.22)	0.114	1.12 (1.02 to 1.23)	0.020

^a^Because this term is part of an interaction term with the cause of injury, it represents the association of injury-care bed density with the dependent variable at the injury cause reference category, which is road traffic injury.

^b^To facilitate the interpretation of the regression coefficients for the cause of injury, bed density was re-coded by subtracting the minimum from each value so that zero in the re-coded variable was in effect the minimum density.

^c^Because this term is part of an interaction term with the injury-care bed density, it represents the association of cause of injury with the dependent variable at the lowest bed density.

^d^Overall p-value for the interaction=0.03.

**Table 3.** Prevalence ratio (95% CI) for the association between injury-care bed density and receiving care from a traditional healer by cause of injury

Cause of injury	All time periods	Earlier than a month ago
Road traffic accident	0.90 (0.85 to 0.96)	0.92 (0.86 to 0.97)
Poisoning	0.99 (0.94 to 1.04)	1.00 (0.95 to 1.06)
Fall	1.00 (0.98 to 1.01)	1.00 (0.99 to 1.02)
Mechanical (non-transport)	1.01 (0.94 to 1.08)	1.02 (0.97 to 1.08)
Fire/hot substance (non-transport)	0.95 (0.91 to 0.996)	0.99 (0.94 to 1.05)
Animal bite/venom	1.00 (0.99 to 1.01)	1.00 (0.99 to 1.02)
Assault	1.00 (0.96 to 1.05)	1.02 (0.96 to 1.02)
Others	0.98 (0.90 to 1.08)	1.02 (0.95 to 1.10)

## Discussion

We sought to uncover factors associated with seeking care from traditional healers after injury in Sudan. We found that educational level, wealth and age were independently associated with receiving care from a traditional healer after injury and that the cause of injury influenced the association of healthcare availability with receiving care from a traditional healer.

The findings mirror what had been discovered about socioeconomic differentials in similar studies of traditional care-seeking for other conditions. The socioeconomic differentials are in agreement with the expectation that traditional healers could be more affordable and therefore are patronised more by those who cannot afford formal healthcare, which is usually associated with user fees. Beyond that, education had an independent association, in line with findings from other studies on other conditions, with no sufficient evidence that it was influenced by wealth or by injury-care bed density. A study in Tanzania also found an association between educational level and the use of traditional medicine.^26^ Our findings could reflect differences by educational level in the experience and level of engagement with health professionals, as well as in health literacy and health belief, all of which can be influenced by educational attainment.^27^ Evidence regarding the use of traditional medicine for infectious diseases and chronic illness suggests that belief about the causes and cures of infectious diseases and chronic illness, as well as in the efficacy and safety of some aspects of traditional medicine, encouraged its use.^10,28–31^ In addition, the decision to visit a traditional healer may not have been the sole responsibility of the injured person or their immediate family; a study among visitors of traditional bone-setters in the capital of the country revealed that relatives and friends played a significant role.^8^

Generally, the evidence linking traditional medicine use with access to formal healthcare in settings like Sudan is very scarce; the study that examined such links in Ghana investigated possible links with possession of health insurance, but found no evidence.^32^ The situation we found was slightly different as we used a different measure and dug deeper into variations by cause of injury. Injury-care bed density is inherently an ecological variable, which may not be an accurate measure of geographical availability and accessibility of injury care at an individual level. In addition, the variable may be associated with other contextual factors, such as the state of road networks, which could independently influence the accessibility of professional health facilities. Such possibilities need to be considered when interpreting the findings. The strongest association was observed when the injury was due to a road traffic accident. Unlike other injuries, by virtue of taking place on roads, the geographical accessibility of healthcare facilities is likely to be higher than with other causes of injury. Moreover, the legal and insurance implications in road traffic accidents demand the involvement of law enforcement, with proper documentation of the consequences of the incident by a healthcare professional. That is achievable only by seeking formal healthcare. Those conditions may explain why seeking care from a traditional healer was responsive to the level of healthcare availability for this category of injury. Injuries due to assault share some, but not all, of the features of road traffic injuries. The different results with injuries due to assault may be attributed to fewer incentives to report assault to official authorities by seeking professional care. A study in Khartoum State showed that almost a third of violent assault cases were perpetrated by a friend or acquaintance.^33^ Attending health facilities in such cases may not be favourable due to the need to file a police report in order to receive care and because of the prevalent tradition to resolve such conflicts internally. It cannot be confirmed, though, if the trend in Khartoum State is generalisable to the national level or is sufficient to explain our finding.

One strength of our analysis is that we tackled uncertainty from differential recall of serious injuries by including the variable on time of occurrence of injury in the multivariable analysis and by running the analysis in a subset of injuries that may have been differentially recalled due to their seriousness. Also, as we used a national survey, our results are fairly generalisable at the national level. However, we note some limitations: we cannot entirely rule out the possibility of social desirability bias, that some people with greater wealth or of a higher educational level may not admit that they went to traditional healers due to the expectations associated with their social standing. The extent of this influence cannot be easily determined given the lack of evidence on stigma associated with traditional healers that is reasonably applicable to our setting. One study from Belgium did not find evidence of a relationship between social desirability and admitting belief in complementary and alternative medicine.^34^ Nevertheless, the plausibility of the uncovered social differentials may counteract the possibility of a substantial role for social desirability in biasing reports of traditional healer use. Another limitation is that we did not have state-specific information on the status of prehospital care or private sector injury-care bed density and therefore could not assess any possible role for those variables. For a similar reason, we could not control for all possible confounders, such as infrastructure factors that enhance geographical accessibility of professional injury care.

### Conclusions

While we cannot confirm causality in the associations observed, our findings allude to some important potential implications. Persistence of education as a determinant of seeking care from traditional healers after injury at all socioeconomic levels asserts the deep-rootedness of the practice. It also implies that improving the affordability of formal healthcare may not translate to lower utilisation of traditional healers. Moreover, merely increasing the availability of injury care facilities in a state may not reduce reliance on traditional healers for all causes of injury. Better educational attainment as a human right and development goal may in the long run alter care-seeking patterns but is outside the remit of the health system. Therefore, taken in the context of the widespread acknowledgement of and experience with the role of traditional healers in supporting the health system, the results support the need to consider potentially suitable roles for traditional healers when developing injury care policies and plans in Sudan. In addition, the role of traditional healers in emergency first aid can be developed with training and support, including guidelines for referral of complex injury and sequential or follow-up care. This is particularly important for those causes of injury where care-seeking patterns may not be responsive to improved availability of professional injury care.

## Supplementary Material

ihz063_Careseeking_at_a_traditional_healer__paper_appendix_revised
