# Supplementary material for: Seeking care from a traditional healer after injury in Sudan: an exploratory cross-sectional analysis
Source: Int Health. 2019 Jul 23;12(3):177–83. doi: 10.1093/inthealth/ihz063 (PMC11973418; doi:10.1093/inthealth/ihz063)
Supplement: ihz063_Careseeking_at_a_traditional_healer__paper_appendix_revised [file inthealth_12_3_177_s3.docx]

Seeking care from a traditional healer after injury in Sudan: exploratory cross-sectional analysis of a national survey

Supplementary table 1a: log prevalence ratios (p-values) from iterations of multivariable models for key variables leading to the set of significant variables (all time periods)

|  | 1 | 2 | 3 | 4 | Final significant variables |
| --- | --- | --- | --- | --- | --- |
| Age (5 year increments) | -0.018 (0.042) | -0.018 (0.041) | -0.018 (0.047) | -0.018 (0.043) | -0.018 (0.054) |
| Gender (ref=male) | -0.065 (0.412) | -0.065 (0.413) | -0.064 (0.413) |  |  |
| Area of residence (ref=urban) | 0.006 (0.964) |  |  |  |  |
| Primary | -0.186 (0.022) | -0.186 (0.022) | -0.185 (0.024) | -0.175 (0.033) | -0.176 (0.034) |
| Secondary + | -0.478 (0.004) | -0.478 (0.004) | -0.479 (0.004) | -0.464 (0.005) | -0.449 (0.007) |
| Wealth score | -0.233 (0.007) | -0.235 (0.001) | -0.233 (0.001) | -0.233 (0.002) | -0.282 (<0.001) |
| Injury-care-related bed density | -0.006 (0.308) | -0.006 (0.312) | -0.006 (0.296) | -0.006 (0.283) |  |
| Poisoning | 0.954 (0.010) | 0.955 (0.010) | 0.953 (0.010) | 0.933 (0.013) | 0.929 (0.013) |
| Fall | 1.607 (<0.001) | 1.608 (<0.001) | 1.610 (<0.001) | 1.599 (<0.001) | 1.604 (<0.001) |
| Mechanical (non-transport) | 1.158 (<0.001) | 1.159 (<0.001) | 1.161 (<0.001) | 1.150 (<0.001) | 1.167 (<0.001) |
| Fire/Hot substance (non-transport) | 0.432 (0.296) | 0.432 (0.295) | 0.432 (0.295) | 0.420 (0.309) | 0.414 (0.320) |
| Animal bite/venom | 1.804 (<0.001) | 1.806 (<0.001) | 1.808 (<0.001) | 1.786 (<0.001) | 1.788 (<0.001) |
| Assault | 0.653 (0.087) | 0.653 (0.088) | 0.653 (0.087) | 0.654 (0.088) | 0.661 (0.085) |
| Others | 0.581 (0.122) | 0.581 (0.119) | 0.580 (0.120) | 0.572 (0.126) | 0.585 (0.117) |
| Time of injury occurrence (ref= 1 month ago) | 0.027 (0.819) | 0.027 (0.818) |  |  |  |

Supplementary table 1b: log prevalence ratios (p-values) from iterations of multivariable models for key variables leading to the set of significant variables (earlier than a month ago)

|  | 1 | 2 | 3 | Final significant variables |
| --- | --- | --- | --- | --- |
| Age (5 year increments) | -0.027 (0.005) | -0.028 (0.005) | -0.028 (0.005) | -0.028 (0.004) |
| Gender (ref=male) | -0.011 (0.897) | -0.011 (0.897) |  |  |
| Area of residence (ref=urban) | -0.020 (0.909) |  |  |  |
| Primary | -0.273 (0.011) | -0.273 (0.011) | -0.271 (0.011) | -0.270 (0.011) |
| Secondary + | -0.379 (0.049) | -0.378 (0.048) | -0.376 (0.049) | -0.379 (0.049) |
| Wealth score | -0.321 (0.002) | -0.316 (<0.001) | -0.316 (<0.001) | -0.297 (<0.001) |
| Injury-care-related bed density | 0.002 (0.712) | 0.002 (0.713) | 0.002 (0.717) |  |
| Poisoning | 0.943 (0.017) | 0.942 (0.017) | 0.938 (0.018) | 0.940 (0.018) |
| Fall | 1.350 (<0.001) | 1.348 (<0.001) | 1.347 (<0.001) | 1.347 (<0.001) |
| Mechanical (non-transport) | 1.073 (0.002) | 1.072 (0.002) | 1.071 (0.002) | 1.066 (0.002) |
| Fire/Hot substance (non-transport) | -0.163 (0.764) | -0.165 (0.761) | -0.167 (0.756) | -0.163 (0.762) |
| Animal bite/venom | 1.557 (<0.001) | 1.554 (<0.001) | 1.550 (<0.001) | 1.551 (<0.001) |
| Assault | 0.294 (0.508) | 0.297 (0.509) | 0.296 (0.509) | 0.294 (0.511) |
| Others | 0.264 (0.529) | 0.263 (0.528) | 0.262 (0.531) | 0.259 (0.533) |
| Time of injury occurrence (ref= 1 month ago) |  |  |  |  |

Supplementary table 2: Interactions terms (first variable*second variable) tested and their rationale

| First variable | Second variable | Rationale |
| --- | --- | --- |
| Injury-care-related bed density | Each of the significant variables | Injury-care-related bed density could influence the associations of some variables; for example, lower density means no access to formal health care for the majority of the population regardless of education or socioeconomic status or cause/severity of injury. |
| Education | Wealth | Being wealthy could modify any association with education because of financial access to formal health care |
| Area of residence | Each of the significant variables | Cultural differences between urban and rural areas may cause some of these associations to vary between urban and rural areas |
| Time of occurrence of injury | Each of the significant variables | Time of occurrence of injury, with different injury severity mix, can also influence some associations, e.g. avoiding traditional healers for severe injuries regardless of wealth or educational level. |

Supplementary table 3: Unadjusted prevalence ratios of key variables for seeking care from a traditional healer in the first week after injury, Sudan Household Health Survey 2010

|  | All time periods | Earlier than a month ago |
| --- | --- | --- |
| Age (5 year increments) | 0.98 (0.96-1.00) | 0.97 (0.95-0.99) |
| Gender (ref=male) | 1.25 (1.05-1.49) | 1.28 (1.04-1.59) |
| Area of residence (ref=urban) | 2.16 (1.68-2.79) | 2.16 (1.59-2.94) |
| Time of injury occurrence (ref= 1 month ago) | 1.15 (0.98-1.48) | NA |
| Educational level (ref = None) |  |  |
| Primary | 0.64 (0.54-0.76) | 0.59 (0.48-0.73) |
| Secondary + | 0.35 (0.25-0.49) | 0.36 (0.25-0.52) |
|  |  |  |
| Wealth score | 0.62 (0.55-0.69) | 0.62 (0.54-0.70) |
|  |  |  |
| Injury-related bed density | 0.97 (0.96-0.98) | 0.98 (0.97-0.99) |
| Cause of injury  (ref = road traffic crash) |  |  |
| Poisoning | 2.80 (1.33-5.91) | 3.05 (1.38-6.73) |
| Fall | 6.65 (3.80-11.65) | 5.26 (2.98-9.28) |
| Mechanical (non-transport) | 4.16 (2.13-8.11) | 3.88 (2.00-7.56) |
| Fire/Hot substance (non-transport) | 2.11 (0.94-4.75) | 1.41 (0.48-4.12) |
| Animal bite/venom | 8.55 (4.91-14.90) | 7.02 (3.99-12.33) |
| Assault | 2.46 (1.15-5.24) | 1.80 (0.75-4.36) |
| Others | 2.35 (1.13-4.89) | 1.66 (0.72-3.87) |

Supplementary table 4:

Adjusted prevalence ratio for care by traditional healer in the first week after injury, SHHS 2010 (final model with significant variables and age as a categorical variable)

|  | Prevalence ratio (95% CI) | p-value |
| --- | --- | --- |
| Age group (ref = 0-5 years) |  |  |
| 5 – 14 years | 1.32 (0.98-1.77) | 0.064 |
| 15 – 44 years | 1.04 (0.76-1.43) | 0.783 |
| 45 – 64 years | 1.09 (0.77-1.53) | 0.635 |
| 65+ years | 0.93 (0.64-1.36) | 0.711 |
| Educational level (ref = None) |  | 0.046 |
| Primary | 0.87 (0.73-1.02) | 0.095 |
| Secondary + | 0.67 (0.48-0.93) | 0.017 |
|  |  |  |
| Wealth score | 0.79 (0.68-0.92) | 0.002 |
|  |  |  |
| Injury-care bed density^a^ centred on minimum ^b^) | 0.90 (0.85-0.96) | 0.001 |
| Cause of injury^c^  (ref = road traffic crash) |  |  |
| Poisoning | 1.02 (0.40-2.62) | 0.968 |
| Fall | 1.75 (0.94-3.25) | 0.077 |
| Mechanical (non-transport) | 1.05 (0.44-2.50) | 0.914 |
| Fire/Hot substance (non-transport) | 0.96 (0.39-2.35) | 0.927 |
| Animal bite/venom | 2.12 (1.21-3.72) | 0.009 |
| Assault | 0.63 (0.25-1.55) | 0.311 |
| Others | 0.71 (0.27-1.86) | 0.489 |
| Interaction^d^ between injury-care bed density and: |  |  |
| Poisoning | 1.10 (1.02-1.19) | 0.016 |
| Fall | 1.11 (1.04-1.18) | 0.001 |
| Mechanical (non-transport) | 1.12 (1.02-1.22) | 0.014 |
| Fire/Hot substance (non-transport) | 1.06 (0.99-1.14) | 0.117 |
| Animal bite/venom | 1.11 (1.05-1.18) | 0.001 |
| Assault | 1.12 (1.04-1.20) | 0.004 |
| Others | 1.10 (0.99-1.22) | 0.087 |

a because this term is part of an interaction term with the cause of injury, it represents the association of injury-care bed density with the dependent variable at the injury cause reference category which is road traffic injury.

b To facilitate the interpretation of the regression coefficients for the cause of injury, bed density was re-coded by subtracting the minimum from each value so that zero in the re-coded variable was in effect the minimum density.

c because this term is part of an interaction term with the injury-care bed density, it represents the association of cause of injury with the dependent variable at the lowest bed density.

d Overall p-value for the interaction = 0.03
